# Artificial Intelligence-Assisted Analysis of Retinal Pigment Epithelium Tears in Patients with Neovascular Age-Related Macular Degeneration Treated with Aflibercept (2 mg and 8 mg) and Faricimab: A Single-Centre Retrospective Case Series

**DOI:** 10.3390/jcm15166478

**Published:** 2026-08-21

**Authors:** Veronika Eggarter, Ludovico Ruscitti, Niccolò Ascioti, Matteo Bonanata, Arianna Peyla, Filippo Simona, Moreno Menghini, Gabriela Grimaldi

**Affiliations:** 1 Department of Ophthalmology, Institute of Clinical Neurosciences of Southern Switzerland (INSI), Ente Ospedaliero Cantonale (EOC), 6962 Lugano, Switzerland; 2 PAX Eye Center, 6600 Locarno, Switzerland; 3 Facoltà di Scienze Biomediche, Università Della Svizzera Italiana, 6900 Lugano, Switzerland

**Keywords:** neovascular age-related macular degeneration (nAMD), retinal pigment epithelium tear, pigment epithelial detachment (PED), anti-VEGF therapy, aflibercept, faricimab, optical coherence tomography (OCT), artificial intelligence, retinal imaging, multimodal imaging

## Abstract

**Purpose**: We aimed to describe retinal pigment epithelium (RPE) tears in patients with neovascular age-related macular degeneration (AMD) and pigment epithelial detachment (PED), focusing on OCT features and AI-assisted image analysis. **Methods**: This retrospective case series included patients with neovascular AMD and PED who developed RPE tears between 2020 and 2025 after treatment with intravitreal aflibercept (2 mg or 8 mg) or faricimab. Multimodal imaging, including spectral-domain OCT and infrared imaging, was reviewed. PED morphology, fluid characteristics, RPE denudation, timing of RPE tear, and visual outcomes were analyzed using manual grading and AI-assisted software. **Results**: Among 375 PED-bearing eyes, 11 eyes of 11 patients developed an RPE tear (2.9%), of which 10 (90.9%) were temporally associated with anti-VEGF treatment. Rates were similar across agents—2.7% (aflibercept 2 mg), 3.0% (aflibercept 8 mg) and 2.4% (faricimab)—with no significant difference. Eight eyes (72.7%) were treatment-naïve and one tear (9.1%) occurred spontaneously after hemorrhage without recent injection. Among treatment-associated tears, 30% followed the first and 50% the second injection (mean 2.1 injections), detected a mean of 44 ± 30 days after the last injection. Mean baseline PED height was 594.8 ± 239.2 μm and median AI-derived PED volume was 2922 nL. Mean RPE-denudation area at detection was 5.25 ± 3.87 mm^2^, remaining stable in 7 of 9 evaluable eyes. Visual acuity was stable or improved in seven eyes; four lost ≥15 ETDRS letters. **Conclusions**: RPE tears were an uncommon, predominantly early complication occurring in eyes with large PEDs, with no excess risk from second-generation agents. AI-assisted OCT may aid anatomical characterisation, and most eyes retained vision with continued therapy.

## 1. Introduction

Retinal pigment epithelium (RPE) tears are a well-recognized complication of neovascular age-related macular degeneration (nAMD), frequently associated with the presence of large fibrovascular or serous pigment epithelial detachments. First described in the literature by Hoskin et al. in 1981 as a possible complication resulting from irregular detachment of the RPE without its basement membrane, the understanding of this condition has progressively evolved over time [1]. Although RPE tears may occur as part of the natural course of large pigment epithelial detachments [2,3] the advent of anti-vascular endothelial growth factor (anti-VEGF) therapy has been associated with an increased recognition of RPE tears [4] and a temporal relationship between intravitreal therapy and tear formation has been suggested. Several authors proposed that the rapid contraction of choroidal neovascular membranes induced by anti-VEGF agents may generate tractional forces that promote tear development [5,6,7]. In their meta-analysis including 17,354 patients from 24 studies, Shi et al. reported an incidence of RPE tears of 1.9% following intravitreal anti-VEGF therapy, although no definitive evidence was found that anti-VEGF treatment itself increases the overall incidence of RPE tears. Furthermore, no significant difference was found in tear incidence among the various anti-VEGF agents [8]. However, current evidence remains largely limited to first-generation anti-VEGF therapies. Pivotal trials on faricimab for nAMD reported a rate of RPE tear of 0.6% [9]. Clemens et al. described in 2023 the first reported case of an RPE tear following intravitreal faricimab injection [10], whereas, to date, no cases of RPE tear after intravitreal treatment with aflibercept 8 mg have been published in the literature.

In parallel with the development of newer anti-VEGF agents, ophthalmology has become increasingly digitalized. Advances in retinal imaging techniques have greatly facilitated the study of RPE tears, particularly through optical coherence tomography (OCT) and fundus autofluorescence imaging, which currently represent the most reliable diagnostic modalities for detecting RPE tears [8,11]. More recently, these imaging modalities have increasingly been analyzed using artificial intelligence (AI)-based approaches, particularly in research settings, with the aim of improving diagnostic accuracy and supporting clinical interpretation [12].

The aim of the present study was to characterise RPE tears occurring during contemporary intravitreal therapy for neovascular AMD, comparing eyes treated with aflibercept 2 mg, aflibercept 8 mg, and faricimab. We focused on multimodal imaging findings and the longitudinal evolution of best-corrected visual acuity, and used AI-assisted OCT segmentation to obtain an objective, three-dimensional characterisation of baseline pigment epithelial detachment morphology.

## 2. Methods

### 2.1. Study Design

**Figure 1:**
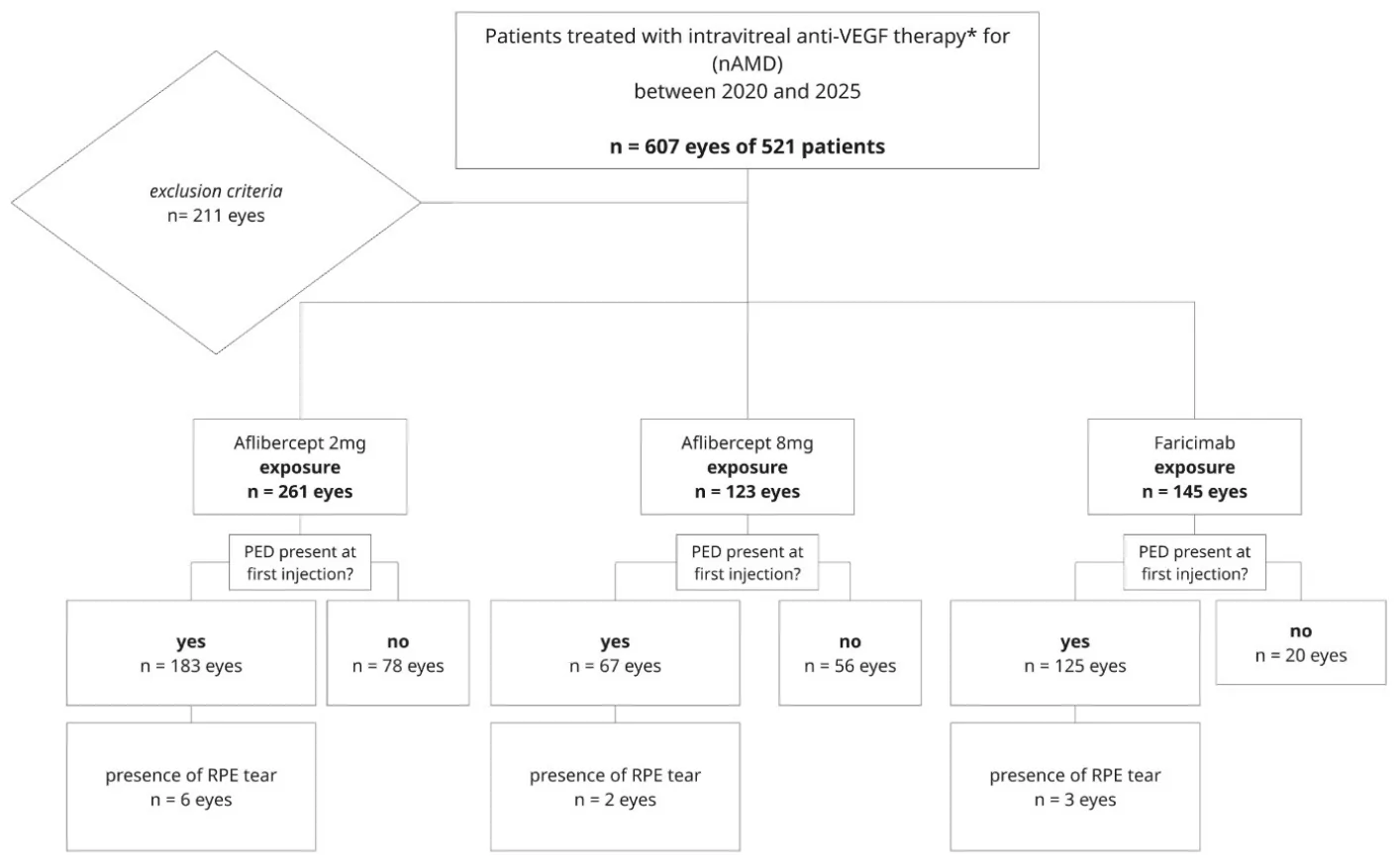
Overview of patient and eye selection. Due to treatment switching during the study period (2020–2025), some eyes were exposed to more than one anti-VEGF agent and were consequently included in multiple treatment groups. * Patients treated exclusively with bevacizumab or ranibizumab were excluded. There were 11 RPE tears in our cohort, including one eye previously treated with aflibercept 2 mg, which was therefore classified within this treatment group in the flowchart, although the tear was not clearly temporally associated with a recent injection. Anti-VEGF: Anti-vascular endothelial growth factor; PED: pigment epithelial detachment; RPE: retinal pigment epithelium.

In this retrospective observational study, all patients treated with aflibercept 2 mg, aflibercept 8 mg or faricimab intravitreal injections for nAMD between 2020 and 2025 at the ophthalmology service of the Ente Ospedaliero Cantonale, Lugano, Switzerland, were identified from the institutional treatment database. Eyes were eligible if they had a baseline pre-treatment OCT scan showing a pigment epithelial detachment (PED) associated with active neovascular disease, and at least 12 months of post-treatment follow-up imaging. Eyes with shorter follow-up were excluded unless an RPE tear occurred before 12 months. Eyes without baseline or follow-up OCT, or with imaging of insufficient quality for grading, were excluded. Eyes were also excluded if diagnosis could not be confidently attributed to neovascular AMD (Figure 1).

Active neovascular AMD was defined by the presence of subretinal and/or intraretinal fluid, subretinal hyperreflective material, hemorrhage and angiographic evidence of macular neovascularization when available. Small drusenoid pigment epithelial detachments without evidence of exudation or neovascular activity were not considered eligible. Among the included patients, eyes with RPE tears were identified and selected for further longitudinal analysis.

The study was conducted in accordance with the tenets of the Declaration of Helsinki and was based exclusively on routinely collected clinical data and imaging. No additional examinations, visits, or imaging procedures were performed for research purposes. Informed consent for the anonymized use of clinical data and imaging for research purposes was available for all included patients (either orally or written).

### 2.2. Outcomes Measured

Demographic data on those with affected eyes, including age at the time of RPE tear occurrence and sex, were recorded. Also, best-corrected visual acuity (BCVA), OCT scans, and corresponding near-infrared (NIR) images of the macula acquired using the Heidelberg Spectralis spectral-domain optical coherence tomography (SD-OCT) platform (Heidelberg Engineering GmbH, Heidelberg, Germany) were analyzed at all predefined time points. One patient was followed-up with after the occurrence of the RPE tear by a local ophthalmologist for logistical reasons. In this case, follow-up imaging was performed using a different device which did not provide an area measurement tool, and image exportation to the RetinAI platform did not yield reliable segmentation results. Therefore, the clinical follow-up of this patient consisted only of BCVA. One patient declined further treatment after the occurrence of the RPE tear and was lost to follow-up for nine years (until 2023); the next available OCT examination was not evaluable because of severe retinal disorganization and extensive subretinal fibrosis, precluding accurate assessment of the RPE-denuded area.

Baseline was defined as the last visit prior to the occurrence of the RPE tear. Additional assessments were collected at the time of tear detection and at 1, 3, 6, 12, 18, and 24 months thereafter. Patients were followed for up to 24 months whenever possible. In cases where a 24-month follow-up visit was unavailable, the examination closest to the 24-month time point was used as the final follow-up assessment. For each affected eye we recorded the anti-VEGF agent administered at the time of the tear, the cumulative number of injections before and after the tear, the treatment regimen (treat-and-extend or pro re nata), and any switching between agents during follow-up.

Several imaging biomarkers were extracted from the available examinations. At baseline, the maximum height of the PED was measured using the integrated measurement tool of the Heidelberg Engineering software (Heidelberg Eye Explorer Version 1.12.1.0, © Heidelberg Engineering). Following the occurrence of the RPE tear, the area of RPE denudation was manually measured on near-infrared (NIR) images at each follow-up visit. In cases of uncertainty, the corresponding OCT B-scans were reviewed to facilitate delineation of the denuded area. All measurements, including PED height and the area of RPE denudation, were performed by a single grader (V.E.). Uncertain cases were reviewed jointly with a second grader (G.G.), and measurements were established by consensus. No disagreements requiring arbitration occurred. The primary grader was not masked to the anti-VEGF treatment received. However, all image analyses were performed according to a predefined measurement protocol to ensure consistency and minimize observer bias.

Baseline OCT scans were included in the AI-assisted analysis only if image quality was sufficient to allow reliable automated segmentation by the RetinAI platform. Volumetric segmentation was successfully performed in 352 of the 375 eyes with PED, including 342 eyes without subsequent RPE tear and 10 of the 11 eyes that developed an RPE tear. The remaining 23 eyes were excluded from the volumetric analysis because of insufficient image quality, accounting for the difference between the denominators used for volumetric and incidence analyses.

Eligible OCT scans were exported in anonymized form and analyzed using the RetinAI Discovery^®^, versions 25-26 (Ikerian AG, Bern, Switzerland). The software automatically segmented the following structures: background, retinal nerve fiber layer, ganglion cell layer and inner plexiform layer, inner nuclear layer and outer plexiform layer, outer nuclear layer, photoreceptors and retinal pigment epithelium, intraretinal fluid, subretinal fluid, pigment epithelial detachment, choriocapillaris, and choroidal stroma. Software-derived volumes of pigment epithelial detachment (PED), intraretinal fluid (IRF), and subretinal fluid (SRF) were extracted for subsequent analysis. Automatic segmentation results were assessed systematically and manual correction of retinal layer segmentation was performed when necessary. The predefined objectives of this manuscript did not include an assessment of manual corrections; their frequency and extent were therefore not systematically documented.

Outcomes of interest included changes in BCVA and in the area of RPE denudation from baseline during follow-up. BCVA was recorded in ETDRS letters and “significant visual loss” was defined as a decrease of ≥15 ETDRS letters.

### 2.3. Statistical Analysis

Owing to the limited sample size and the exploratory nature of the study, statistical analyses were primarily descriptive. Continuous variables were reported as mean ± standard deviation (SD) or median and interquartile range (IQR), as appropriate, whereas categorical variables were summarized as frequencies and percentages. Descriptive calculations were performed in Microsoft Excel^®^ (Microsoft Corporation, Redmond, WA, USA; Version 2408). Inferential statistical testing was performed in R version 4.6.1 (R Foundation for Statistical Computing, Vienna, Austria). The Fisher–Freeman–Halton exact test was used to compare the proportion of eyes developing RPE tears among treatment groups; baseline PED volume was compared across the three treatment groups using the Kruskal–Wallis test and between eyes that did and did not develop a tear using the Mann–Whitney U test. A two-sided *p*-value < 0.05 was considered statistically significant.

## 3. Results

All patients with nAMD undergoing intravitreal anti-VEGF therapy were screened (*n* = 521). Treatment data comprised 452 eyes treated with aflibercept 2 mg, 137 eyes treated with aflibercept 8 mg, and 149 eyes treated with faricimab. After application of the exclusion criteria, the total cohort comprised 338 patients, who could contribute one or both eyes. Because some patients were treated bilaterally, and because individual eyes could be switched between anti-VEGF agents during the study period, a single eye could contribute to more than one treatment category. The unit of analysis was therefore the treatment exposure, defined as the combination of one eye and one anti-VEGF agent, rather than the eye itself. Accordingly, the total number of exposures exceeds the number of eyes, and the comparisons between agents presented below should be interpreted as exposure-based rather than eye-based.

After application of the inclusion and exclusion criteria, 211 eyes were excluded from further analysis (Figure 1). The remaining eyes were stratified according to the presence or absence of PED at the initiation of intravitreal therapy or, in eyes that underwent treatment switching, at the time of switching to a different anti-VEGF agent. For clarity, all analyses and results are reported at the eye level rather than the patient level. Eyes without a PED present at time of first injection were not subjected to further detailed analysis and were only monitored for the occurrence of RPE tears during follow-up.

During follow-up, RPE tears developed in 11 eyes of 11 patients, 36.4% of those were men, 63.6% women. The mean age at time of RPE tear was 83.2 years ± 7 years (range: 66–90 years). Demographic and further clinical details for the affected patients are shown in Table 1 and Table 2. In 10 of these cases (90.9%), the event occurred in close temporal association with intravitreal anti-VEGF treatment. At the time of tear occurrence, 5 patients were receiving aflibercept 2 mg, 2 patients aflibercept 8 mg, and 3 patients faricimab. Among eyes receiving anti-VEGF therapy, treatment-associated RPE tears occurred in 2.7% of eyes treated with aflibercept 2 mg who presented a PED at beginning (*n* = 183), 3.0% of eyes treated with aflibercept 8 mg (*n* = 67), and 2.4% of eyes treated with faricimab (*n* = 125). The treatment-associated prevalence of RPE tear was 2.7% (*n* = 10/375, 95% CI 1.3–4.9%). No evidence of a difference in the risk of RPE tears was observed among the treatment groups (Fisher–Freeman–Halton exact test, *p* = 0.95). However, given the small number of events, the study was underpowered to exclude clinically meaningful differences between treatments.

One additional patient developed an RPE tear following an acute macular hemorrhage 210 days after the last aflibercept 2 mg injection while being managed under a pro re nata (PRN) treatment regimen. As the event was considered unrelated to recent anti-VEGF treatment and no appropriate baseline visit was available, this patient was excluded from analyses of treatment-associated RPE tears and from analyses requiring baseline data.

At baseline, the mean best-corrected visual acuity (BCVA) of the affected patients was 60 ± 16 ETDRS letters. Baseline OCT imaging demonstrated PEDs with a mean maximum height of 594.8 μm ± 239.2 μm. AI-assisted segmentation was successfully completed in all eyes except the one with spontaneous RPE tear (Patient 5), where imaging was not available, and enabled quantification of PED and fluid volumes. Among PED-bearing eyes that did not develop a tear, baseline PED volume was comparable across treatment groups: a median of 266 nL (IQR 118–649; *n* = 158) for aflibercept 2 mg, 276 nL (IQR 125–519; *n* = 65) for aflibercept 8 mg, and 273 nL (IQR 150–760; *n* = 119) for faricimab, with no significant difference (Kruskal–Wallis, *p* = 0.67). By contrast, eyes that developed a treatment-related RPE tear had markedly larger baseline PEDs (median 2922 nL, IQR 1732–4731; *n* = 10) than PED-bearing eyes that did not tear (median 275 nL, IQR 122–669; *n* = 342), with an approximately elevenfold difference (Mann–Whitney U, *p* < 0.001).

Among the 10 patients with treatment-associated RPE tears, 9 tears (90%) occurred in the loading phase (mean of 2.1 injections preceding the event). The tears were detected at a mean of 44 ± 30 days after the last anti-VEGF injection. One RPE tear (10%) occurred after the 6th injection.

**Figure 2:**
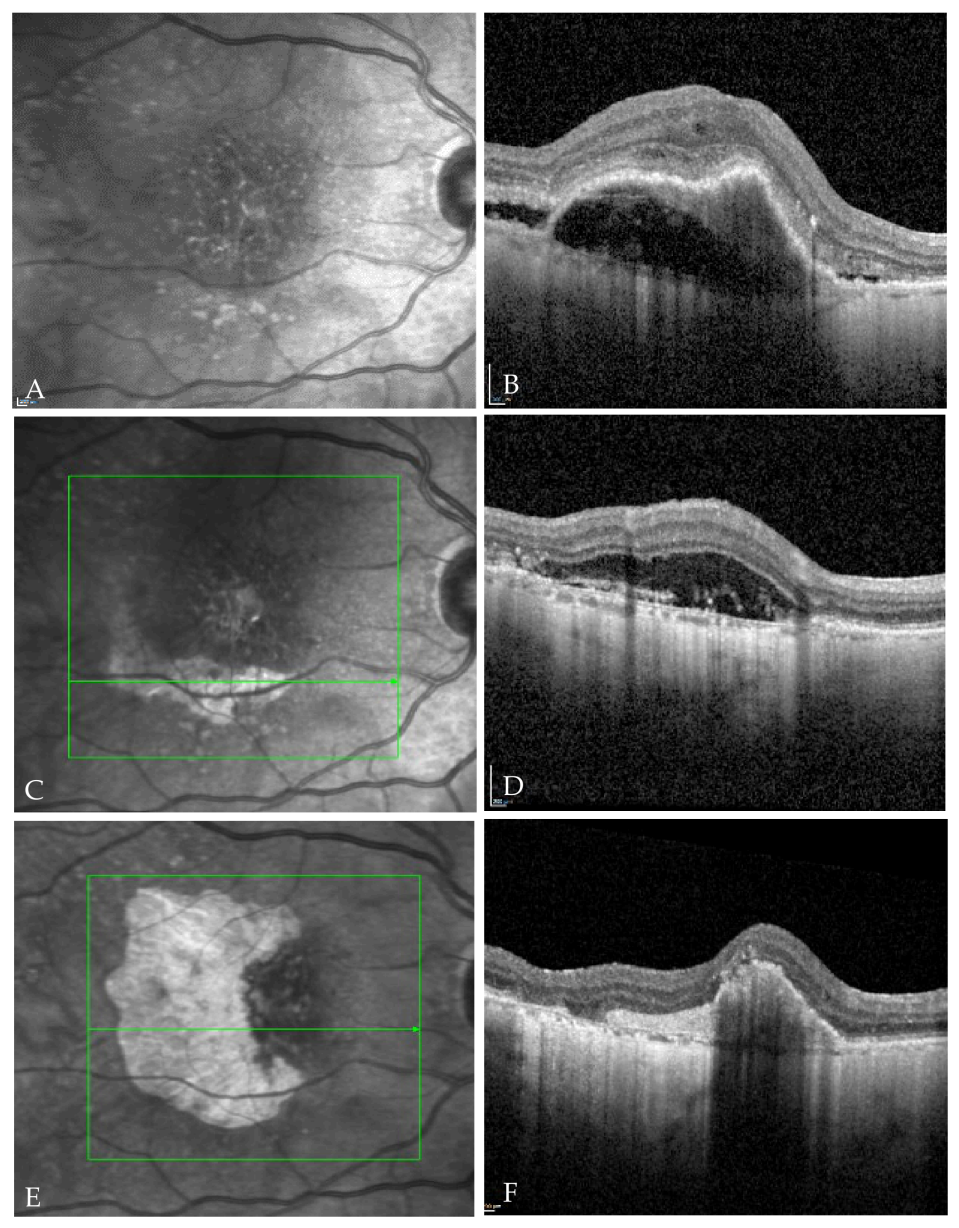
Patient 10. Multimodal imaging showing progression of retinal pigment epithelium (RPE) denudation area. Near-infrared reflectance images (**A**,**C**,**E**) and corresponding optical coherence tomography (OCT) scans (**B**,**D**,**F**) demonstrate progressive enlargement of the RPE-denuded area. (**A**,**B**) Baseline imaging prior to the first intravitreal injection (IVI). (**C**,**D**) Imaging after the first IVI at the time of RPE tear detection. (**E**,**F**) Follow-up imaging one year after RPE tear occurrence.

**Figure 3:**
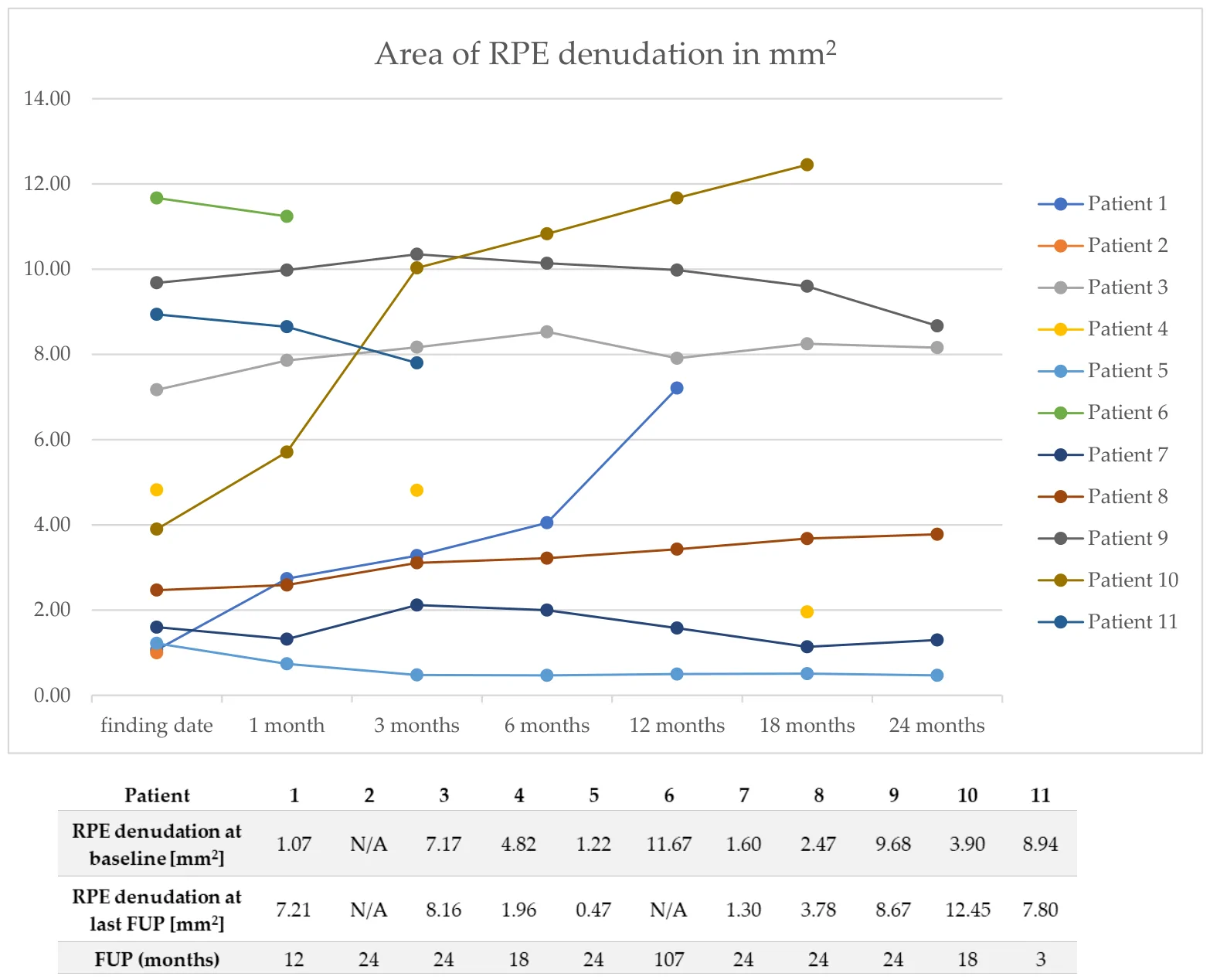
Progression of the area in mm^2^ of retinal pigment epithelium (RPE) denudation after tear occurrence. Patient 2 was excluded from this analysis because follow-up was performed by the referring ophthalmologist, precluding standardized assessment of the area of RPE denudation. In Patient 6, who was lost to follow-up for 9 years, the first available OCT examination after re-presentation was not evaluable because of advanced subretinal fibrosis. N/A: not available; FUP: follow-up.

The mean area of RPE denudation at the time of tear detection was 5.25 ± 3.87 mm^2^. During follow-up, the area of RPE denudation remained stable in 7 of 10 evaluable eyes (70%). One eye (Patient 2) was excluded from this analysis because follow-up was performed by the referring ophthalmologist, precluding standardized assessment of lesion area. Marked enlargement of the denuded area was observed in 2 of 10 eyes (20%). Representative multimodal imaging illustrating progression of RPE denudation is shown for Patient 10 in Figure 2, while the longitudinal changes for all affected eyes are summarized in Figure 3.

**Figure 4:**
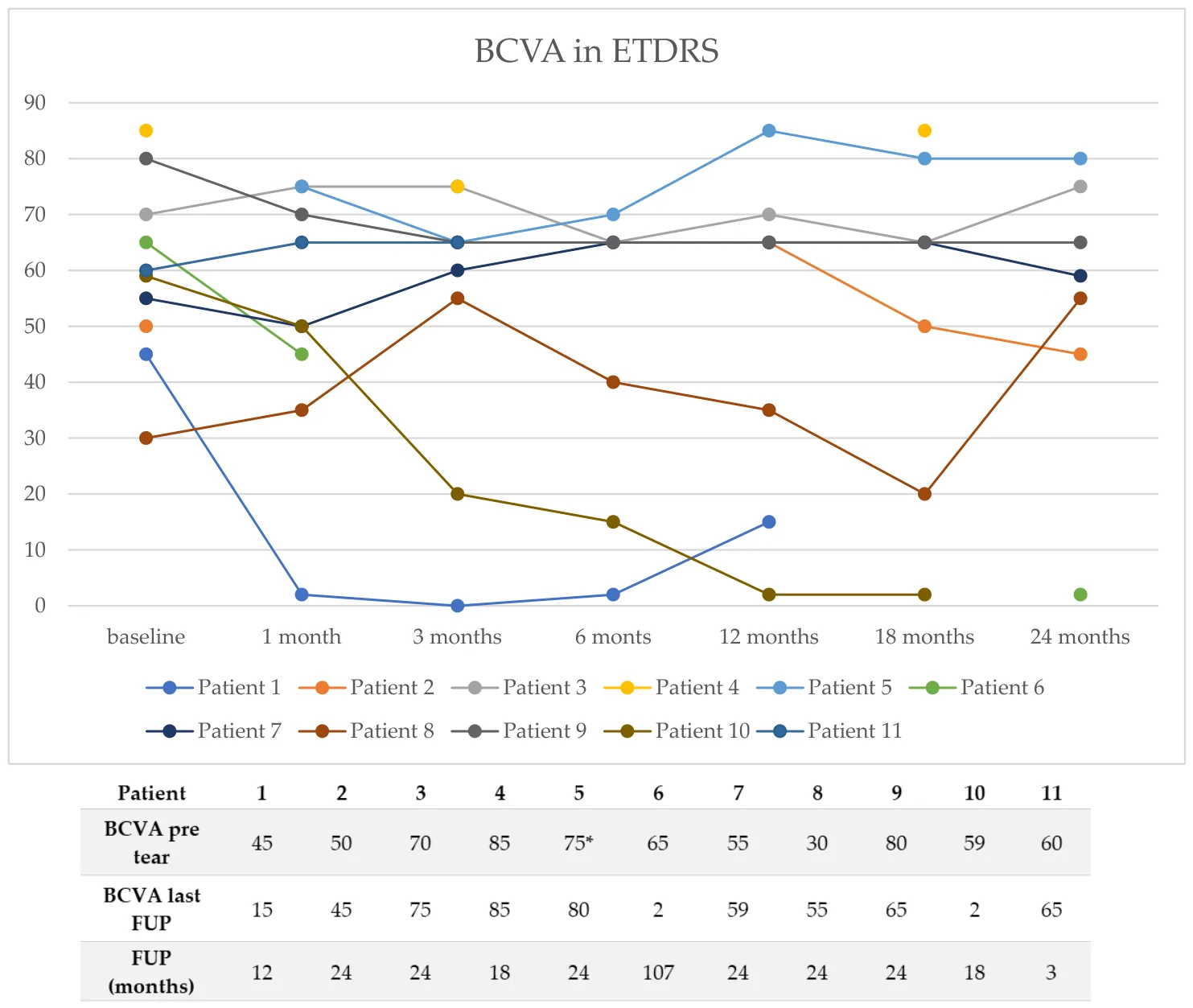
Progression of best-corrected visual acuity (BCVA) in affected eyes. Note: Patient 2 developed a cataract in the affected eye during follow-up. Patient 8 underwent cataract surgery after 18 months of follow-up. Patient 6 was lost to follow-up for 9 years; therefore, the BCVA value at 24 months corresponds to the first available follow-up examination. * Patient 5 was excluded from the baseline BCVA analysis because the RPE tear occurred spontaneously in association with an acute macular hemorrhage, and no suitable pre-tear baseline examination was available. For this patient, the reported pre-tear BCVA corresponds to the BCVA measured at the time of RPE tear detection. BCVA: Best corrected visual acuity; FUP: follow-up.

Visual outcomes were heterogeneous. Four of eleven patients (36.4%) experienced a significant loss of visual acuity, defined as a decrease of at least 15 ETDRS letters. In contrast, seven patients (63.6%) demonstrated either stable or improved BCVA despite the occurrence of an RPE tear. One patient (Patient 8) underwent cataract surgery during follow-up, which may have contributed to the observed improvement in BCVA. Conversely, another patient (Patient 2) developed a cataract during the observation period but underwent surgery only after completion of follow-up; therefore, the cataract may have contributed to the observed reduction in BCVA. Further details are shown in Figure 4. Among all affected eyes (11 eyes), the mean follow-up duration after RPE tear occurrence was 850 days (range, 78–3282 days). Anti-VEGF therapy was continued in 10 of 11 eyes after the tear; one patient declined further treatment. Switching between agents occurred in several eyes during follow-up, but the occurrence of a tear rarely led to permanent discontinuation, and long-term therapy remained feasible in most affected eyes.

## 4. Discussion

This retrospective observational study analyzed patients undergoing intravitreal anti-VEGF therapy for nAMD at a single tertiary referral center. In addition to conventional clinical and imaging assessments, OCT images at baseline were analyzed using an AI-based segmentation platform. The study provides a real-world characterization of RPE tears occurring during treatment with both first- and second-generation anti-VEGF agents.

The 2.4–3.0% rate of treatment-associated RPE tears in our cohort fits well in the reported range and aligns closely with the 2.3–3.2% reported by Vazquez-Alfageme et al. for ranibizumab and aflibercept and with the 1.9% pooled incidence of Shi et al. [4,8]. It is notably lower than the 15–25% described in cohorts selected for large or vascularised PEDs—for example 25% in the prospective RECOVER study of monthly ranibizumab for vascularised PED [13], and roughly 20% among ‘giant’ PEDs (>550 μm) in the Vazquez-Alfageme series [4]—and far higher than the 0.2–0.25% recorded across unselected VIEW 1–2 eyes [14]. This wide spread reflects how strongly incidence depends on case-mix, in particular on PED size and morphology, and suggests that our denominator of all PED-bearing eyes, rather than only voluminous PEDs, may account for the comparatively low rate. Crucially, and consistent with Shi et al. [8], we found no signal of increased risk with the second-generation agents aflibercept 8 mg or faricimab, for which comparative data remain sparse. The introduction of more potent anti-VEGF therapies has indeed raised concerns that rapid and enhanced flattening of PEDs could increase the risk of RPE tears through enhanced contraction of the underlying neovascular complex. In our cohort, no apparent increase in risk was observed with second-generation agents, although definitive conclusions cannot be drawn from the present sample size.

**Figure 5:**
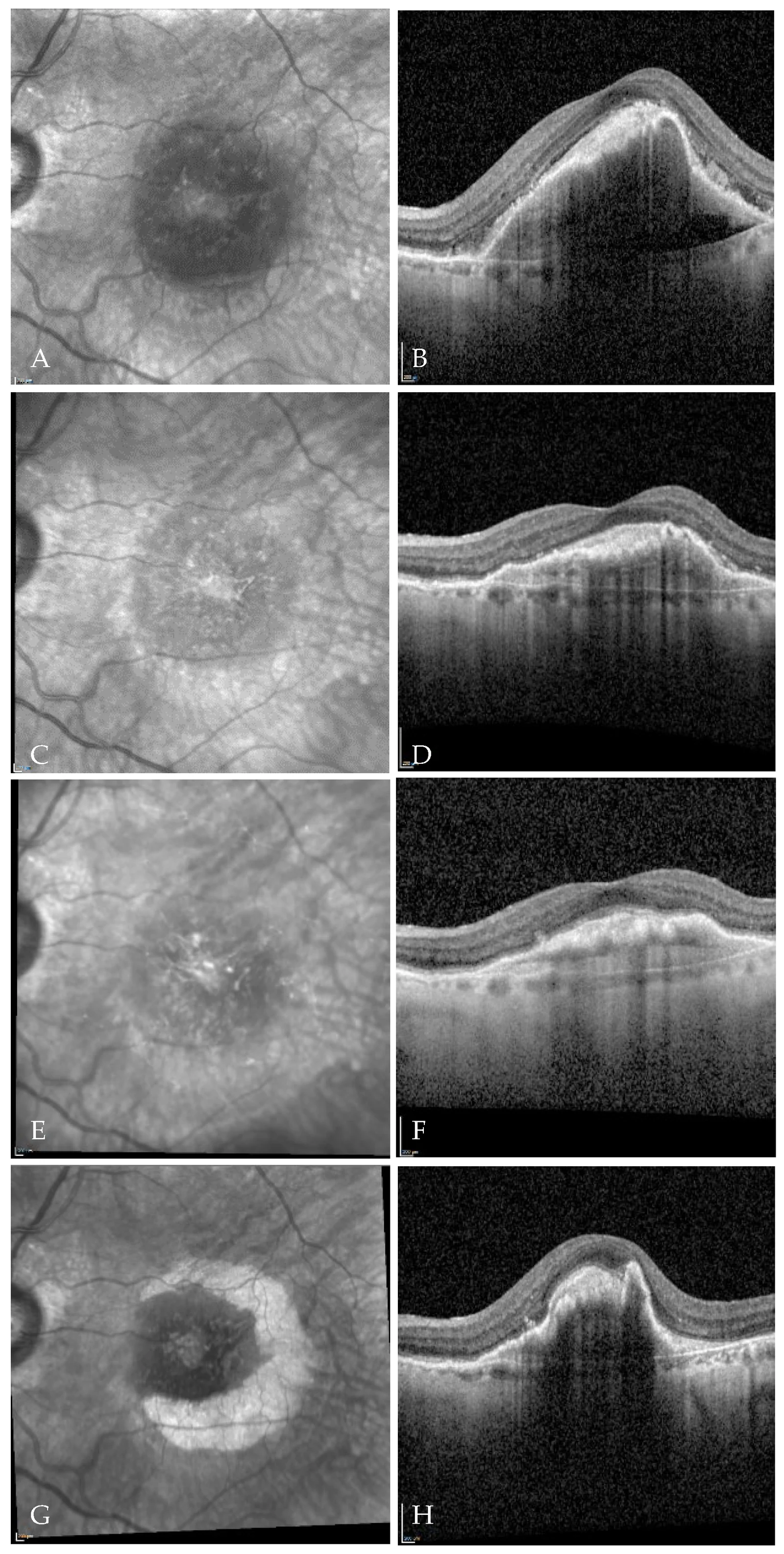
Patient 11. Multimodal imaging demonstrating the temporal evolution of pigment epithelial detachment (PED) changes. Optical coherence tomography (OCT; (**B**,**D**,**F**,**H**)) and near-infrared reflectance (NIR; (**A**,**C**,**E**,**G**)) images are shown at different time points. (**A**,**B**) Prior to the first intravitreal injection (IVI) of aflibercept 2 mg. (**C**,**D**) After the first IVI of aflibercept 2 mg. (**E**,**F**) Prior to the sixth IVI of aflibercept 8 mg. (**G**,**H**) After the sixth IVI of aflibercept 8 mg.

The temporal pattern in our series mirrors the literature. Tears were detected a mean of 44 days after the last injection, with most occurring after the first or second injection, closely matching the 55 days and 1.8 injections of Vazquez-Alfageme et al., in whose cohort 82.6% of tears arose between the first and second injection, and the loading-phase clustering reported by Durkin et al. Invernizzi et al. refined this picture by distinguishing ‘early’ tears (before ~182 days, ~70% of cases) from ‘late’ tears, proposing that early tears arise from anti-VEGF-induced contraction of a responsive neovascular complex, whereas late tears reflect progressive disease in poorly responding eyes [4,15,16]. Our cohort recapitulates both phenotypes: the majority were early, loading-phase events, while one eye tore only after the sixth injection of aflibercept 8 mg following escalation from aflibercept 2 mg (Patient 11). This late, post-switch tear, occurring after substantial PED flattening, fits the late-tear pattern and supports the notion that long-term mechanical stress, rather than acute membrane contraction alone, can ultimately compromise RPE integrity. The clinical course of this patient is illustrated in Figure 5.

The presence of a large PED was the dominant anatomical feature in our series, consistent with the literature, in which PED height thresholds of 400–600 μm have repeatedly been associated with tear risk [5,17,18,19], and Nagiel et al. described a steep rise in RPE stretching above ~600 μm [20]. Our mean baseline height of 594.8 μm falls squarely within this high-risk band. The added value of AI-assisted segmentation was the objective, three-dimensional quantification of PED volume alongside height. Tear rates across agents were comparable and not attributable to differing PED burden, but eyes that subsequently tore had baseline PED volumes roughly an order of magnitude larger than those that did not (median 2922 vs. 275 nL; *p* < 0.001), confirming PED volume as a strong correlate of tear occurrence. This separation was clear at the group level, yet the wide overlap of individual values and the absence of a discrete cut-off mean that volume alone does not reliably predict a tear in a given eye, echoing the conclusion of Ersoz et al. and Khanani et al. [11,21] that tear formation is multifactorial, involving CNV/PED ratio and microstructural RPE changes [13,22] that our retrospective dataset could not capture.

Given the absence of clearly defined risk thresholds, early detection and treatment of high-risk PEDs remain important clinical objectives. Preventing progression to large PEDs may reduce the risk of subsequent RPE tears and associated vision loss. Equally important is appropriate patient counseling. Individuals presenting with large PEDs should be informed that RPE tears may occur as part of the natural disease course or during anti-VEGF treatment. Such counseling may help maintain treatment adherence should this complication occur.

Visual outcomes were heterogeneous but, on balance, reassuring: seven of eleven eyes maintained or improved vision despite the tear. This accords with the consistent message across the literature, from Vazquez-Alfageme’s positive correlation between injection number and visual outcome, to Khanani et al.’s conclusion that vision usually stabilises with continued therapy, to Invernizzi et al.’s key insight that long-term acuity is governed more by an eye’s underlying response to treatment than by the tear itself. Our data reinforce that continued anti-VEGF therapy after a tear is both feasible and worthwhile in eyes with ongoing exudation, and that, as Durkin et al. and others have shown, subfoveal involvement and subsequent disciform scarring, rather than the tear per se, drive the poorest outcomes [4,15,16,21,23].

This study has several limitations. Its retrospective design may have introduced selection and information bias, and the number of RPE tears was small, limiting statistical power and precluding robust comparisons among treatment groups. Furthermore, this was a single-center study, which may limit generalizability. Nevertheless, the rarity of RPE tears makes prospective studies difficult to conduct, and carefully documented case series remain an important source of clinical evidence. The use of multimodal imaging combined with AI-assisted OCT analysis allowed objective characterization of anatomical features associated with RPE tears and provides additional data regarding this uncommon but clinically relevant complication in the era of contemporary anti-VEGF therapy. However, because PED volume and maximum PED height are closely correlated, our findings cannot determine whether AI-derived volumetric measurements provide predictive information beyond conventional OCT parameters. Moreover, the current AI platform was limited to volumetric assessment and did not generate additional morphologic biomarkers that may be relevant for estimating the risk of RPE tear formation. Given the small number of eyes with RPE tears and the absence of multivariable analyses, these observations should be considered exploratory and hypothesis-generating rather than evidence of validated predictive biomarkers. Future studies with larger cohorts are warranted to determine whether AI-assisted quantitative imaging can improve risk stratification beyond established clinical and OCT-based parameters.

In this single-centre series characterising RPE tears across first- and second-generation anti-VEGF agents, tears were an uncommon complication (2.4–3.0% of PED-bearing eyes) that clustered in the loading phase and occurred predominantly in eyes with large PEDs, with no apparent excess risk from aflibercept 8 mg or faricimab. AI-assisted OCT enabled objective volumetric characterisation of these detachments. Most eyes retained useful vision when anti-VEGF therapy was continued. Larger, multicentre datasets, ideally combining quantitative imaging biomarkers with longitudinal follow-up, will be needed to define which eyes are genuinely at risk and to confirm the safety profile of newer high-dose agents.

## Figures and Tables

**Table 1:** Baseline and demographic data. PED: Pigment Epithelial Detachment; SRF: subretinal fluid; IRF: intraretinal fluid; OCT: Optical Coherence Tomography; N/A: not available. Data are partially missing for Patient 5 because no appropriate baseline visit was available; this patient developed their RPE tear following an acute macular hemorrhage.

Patient	Laterality	Age at Baseline	Gender	Naive/Switcher	PED Volume Baseline [nL]	SRF Baseline [nL]	IRF Baseline [nL]	Height PED OCT [μm]
1	RE	85	M	N	680	1	136	290
2	LE	80	F	N	7205	1563	70	1056
3	LE	83	F	N	5351	1762	<1	746
4	LE	66	M	N	3222	470	<1	666
5	LE	90	F	-	N/A	N/A	N/A	N/A
6	LE	76	M	N	2163	115	<1	608
7	RE	88	F	N	958	1735	<1	453
8	LE	88	F	N	5230	1086	570	776
9	RE	85	F	S	2621	374	<1	484
10	RE	85	M	N	3234	913	15	614
11	LE	89	F	S	1589	<1	<1	255

**Table 2:** Clinical data on tear occurrence. A 2 mg: Aflibercept 2 mg; A 8 mg: Aflibercept 8 mg; Far: Faricimab; ETDRS: Early Treatment Diabetic Retinopathy Study; RPE: Retinal Pigment Epithelium; IVI: intravitreal injection; FUP: Follow-up visit; N/A: not available. * RPE tear occurred during the loading phase, although the exact injection number could not be established due to the lack of interim imaging. Foveal involvement by RPE denudation was assessed on the corresponding near-infrared (NIR) image using the ETDRS grid, with involvement of the central 1-mm subfield considered indicative of foveal involvement. Patient 2 was excluded from this analysis because follow-up was performed by the referring ophthalmologist elsewhere, precluding standardized assessment of the area of RPE denudation. In Patient 6, who was lost to follow-up for 9 years, the first available OCT examination after re-presentation was not evaluable because of advanced subretinal fibrosis.

Patient	Days to RPE Tear After IVI [d]	IVI Number	IVI Medication	Area of RPE Denudation at Time of Tear Detection [mm^2^]	24 Months or Last Available Follow-Up [Months After RPE Tear]	Central 1 mm of ETDRS Grid Involved in RPE-Denudation Area at 24 m or Last FUP
1	29	1	A 2 mg	1.07	12	N
2	111	unsure *	A 2 mg	N/A	24	N/A
3	28	2	A 2 mg	7.17	24	N
4	21	2	A 2 mg	4.82	18	N
5	-	-	-	1.22	24	N
6	32	2	A 2 mg	11.67	107	N/A
7	32	1	Far	1.60	24	N
8	56	2	Far	2.47	24	N
9	56	2	Far	9.68	24	N
10	7	1	A 8 mg	3.90	18	Y
11	70	6	A 8 mg	8.94	3	N

## Data Availability

The datasets generated and/or analysed during the current study are available from the corresponding authors on reasonable request.

## Abbreviations

(n) AMD	(neovascular) age-related macular degeneration
RPE	retinal pigment epithelium
PED	pigment epithelial detachment
anti-VEGF	anti-vascular endothelial growth factor
BCVA	best-corrected visual acuity
OCT	optical coherence tomography
SD-OCT	spectral-domain optical coherence tomography
NIR	near-infrared reflectance imaging
SRF	subretinal fluid
IRF	intraretinal fluid
IVI	intravitreal injection
ETDRS	Early Treatment Diabetic Retinopathy Study
PRN	pro re nata
AI	artificial intelligence
SD	standard deviation
IQR	interquartile range
FUP	follow-up
N/A	not available
